# The Prevalence and Associated Factors of Attention Deficit Hyperactivity Disorder Among Primary School Children in Amman, Jordan

**DOI:** 10.7759/cureus.37856

**Published:** 2023-04-19

**Authors:** Layali N Abbasi, Tarek Mazzawi, Lamees Abasi, Sara Haj Ali, Abdallah Alqudah, Hasanen Al-Taiar

**Affiliations:** 1 Medicine, Al-Balqa Applied University, Al-Salt, JOR; 2 Pathology and Laboratory Medicine, King Hussein Medical Center, Royal Medical Services, Amman, JOR; 3 Medicine, University Hospitals Plymouth NHS Trust, Plymouth, GBR; 4 Psychiatry, Oxford Health NHS Foundation Trust, Oxford, GBR

**Keywords:** schoolchildren, conners, prevalence, child psychiatry, adhd

## Abstract

Objective

Attention deficit hyperactivity disorder (ADHD) is a common neurodevelopmental disorder characterized by impaired levels of inattention, disorganization and/or hyperactivity-impulsivity. The aim of this study was to estimate the prevalence of ADHD among primary school children in Jordan and assess the potential risk factors.

Method

A cross-sectional study was conducted in 2022-2023 on 1563 school children aged six to 12 years. ADHD was assessed using parent and teacher versions of the Conners Rating scale. Risk factors were evaluated through a sociodemographic questionnaire. A p-value set at <.05 was considered statistically significant.

Results

ADHD prevalence based on parents' and teachers' perspectives was 27.7% and 22.5%, respectively. Males, smoking during pregnancy, low birth weight, low parental education and unemployment, and public schools had increased ADHD rates.

Conclusion

ADHD presents a major problem among primary school children in Jordan. Early detection, prevention, and management of this disease require parents' and teachers' awareness and risk factor control.

## Introduction

Attention deficit hyperactivity disorder (ADHD) is characterized by a pattern of inattention, disorganization, and/or hyperactivity-impulsivity with impaired functioning in academic, social, or occupational activities [1]. It is a prevalent neurodevelopmental disorder associated with poor scholastic performance and outcomes [1]. According to population surveys, the worldwide prevalence of ADHD in children is 7.2% [1,2]. ADHD is a chronic condition that starts in childhood [3] and lasts into adulthood, with an average rate of 43% [4]. 

The Diagnostic and Statistical Manual of Mental Disorders fifth edition (DSM-5) lists a number of symptoms of ADHD, including a failure to pay close attention to details, difficulty sustaining attention, not listening when spoken to directly, failure to finish schoolwork, difficulty organizing tasks, reluctant to engage in tasks that require sustained mental effort, losing things necessary for tasks, easily distracted, forgetful in daily activities, fidgeting, leaving the seat in situations when remaining seated is expected, unable to play quietly, acting as if "driven by a motor", talking excessively, difficulty waiting in turn and interrupting others at rates that are disproportionate to the person's age or stage of development [1,5]. These symptoms should be present in at least two settings (school and home) and before the age of 12 years [1]. 

ADHD is the most common neurodevelopmental disorder in children [6]. Studies on the prevalence of ADHD among children in Arab countries are reportedly sparse and provide scant data. In the last 25 years, 26 studies on the prevalence of ADHD have been conducted in Arab countries, with rates ranging widely from 1.4% to 34.5%, according to a recent analysis [7,8].

The etiology of ADHD is multifactorial, involving both genetic and environmental variables [9]. ADHD has been linked to a variety of risk and adversity factors; however, the majority of these links have yet to be shown as causal [10].

Patients with ADHD often also suffer from co-occurring mental health issues or comorbidities, such as depression, anxiety, a learning disability, or a behavioral condition, such as conduct disorder or oppositional defiant disorder. Moreover, they are more likely to engage in dangerous activities like substance abuse, delinquency, and traffic accidents, making ADHD a serious public health issue [11,12].

In Jordan, the prevalence of ADHD in schoolchildren has not been previously studied from the perspective of both parents and teachers. This study aimed to determine the prevalence of ADHD among school-aged children in Jordan, as well as potential risk factors associated with this condition.

This article was posted as a preprint on Research Square on 2/24/2023. 

## Materials and methods

A cross-sectional descriptive study was conducted at public and private primary schools in Amman, the capital of Jordan, during the study year of 2022-2023. Children of both genders aged six to 12 years old were included in the survey. 

After approval from Al-Balqa Applied University's Institutional Review Board (IRB number: 26/3/1/944) and the Ministry of Education (approval number: 7/13), a psychologist visited the schools to explain the study's objectives and methodology to the principals in order to obtain their permission to distribute the questionnaires. Information was gathered for the study by self-administered questionnaires. The questionnaire for parents is divided into two parts. The child's age, gender, and health conditions, as well as the parents' occupation and level of education, were among the demographic details and potential factors uncovered in the first section. In the second part, parents and teachers used the Arabic version of a 10-item Conners Rating Scale for ADHD to evaluate the child. Teachers and parents were informed that all data collected for the study would be kept confidential and used exclusively for research. The classroom teacher filled out a questionnaire about each student, and the parents of those students filled out another questionnaire that was sent home in their child's backpack.

The sampling plan was stratified and proportionally allocated to ensure a representative sample of the research population. Eight elementary schools were chosen randomly. Using a simple random method, classrooms from these schools were selected at random. All students who were present in these selected classrooms were included in the study. A total of 2400 students were approached, and 1563 agreed to participate in the study, with a response rate of 65%. Due to incomplete questionnaires, almost a quarter of the sample was excluded from the study.

The 10-item Conners Rating Scale comprises the 10 items with the highest loading from the original Conners Parent and Teacher Rating Scales, along with updated normative data [13]. Each item was evaluated on a four-point scale ranging from zero to three to reflect how often the behavior is noted; (0 = not at all, 1 = a little, 2 = much, and 3 = very much) with a total of 30 points, a score of 15 or higher indicates a high risk for ADHD [14,15]. 

Data analysis was performed using the SPSS statistics program (version 28.0; IBM Inc., Armonk, New York). Percentage and frequency were used for categorical variables. Chi-squared test was used to examine the association between sociodemographic variables and ADHD. Multiple binary logistic regression analysis was performed to assess ADHD predictors. The intraclass correlation and Cohen's kappa were used to investigate parent-teacher agreement. A p-value of <.05 was considered statistically significant.

## Results

Our study included 1563 children, who ranged in age from six to 12 years old. Of these, 770 were boys (49.0%), and 790 were girls (51.0%). The sociodemographic characteristics and potential risk factors are described in Table (1)

**Table 1:** Study participants' sociodemographic characteristics and potential risk factors (n=1563)

Variable	Categories	Frequency	Percentage
Gender	Male	770	49.0
	Female	790	51.0
School type	Public	857	54.8
	Private	706	45.2
Age (years)	6	212	13.6
	7	292	18.7
	8	295	18.9
	9	221	14.1
	10	159	10.2
	11	210	13.4
	12	174	11.1
Father's education	Secondary	138	8.9
	High school	389	24.8
	Postgraduate	1036	66.3
Mother's education	Secondary	101	6.4
	High school	411	26.3
	Postgraduate	1051	67.3
Father's occupation	Employed	711	45.5
	Unemployed	852	54.5
Mother's occupation	Employed	533	34.1
	Unemployed	1030	65.9
Smoking during pregnancy	No	1534	98.1
	Yes	29	1.9
Neonatal disorders	No	1524	97.5
	Yes	39	2.5
Birth weight	Low	257	16.4
	Normal	1268	81.1
	High	38	2.5
Season of birth	Spring	426	27.3
	Summer	394	25.2
	Fall	385	24.6
	Winter	358	22.9

Prevalence of ADHD and associated factors

In our study, the prevalence of ADHD, according to parents' perspective, was 27.7%, and according to teachers' perspective was 22.5%. 

Regarding associated factors, males were substantially more likely than females to have ADHD (31.2% versus 24.3%, p=0.003). In public schools, 32.3% of students had ADHD compared to 22.1% in private schools (p<0.001). In addition, low parental education was found to be significantly associated with ADHD (<0.001 for fathers and 0.002 for mothers). 

The prevalence of ADHD among children of unemployed fathers (32%; p<0.001) and unemployed mothers (29.3%; p=0.047) was shown to be statistically significant. The study indicated that 51.7% of mothers who smoked during pregnancy had a child with ADHD, compared to 27.2% of non-smoking mothers (p=0.004). In addition, a significant proportion of children with ADHD had low birth weight (p<0.001). In contrast, neonatal diseases and season of birth had no significant connection with ADHD. 

Table 2 displays the Chi-square distribution of probable sociodemographic variables and other risk factors associated with ADHD, as rated by parents.

**Table 2:** Chi-squared distribution of ADHD potential sociodemographic variables and other risk factors according to the parents' rating scale (n=1563) ADHD - attention deficit hyperactivity disorder

Variable		Without ADHD	With ADHD	x2 Chi-squared	p-value
Gender	Male	530 (68.8%)	240 (31.2%)	9.101	0.003
						Female	600 (75.7%)	190 (24.3%)
	School type	Public	580 (67.7%)			277 (32.3%)	20.210	<0.001
Private		550 (77.9%)	156 (22.1%)					
Father's education	Secondary	88 (63.8%)	50 (36.2%)	15.204	<0.001
	High school	261 (67.1%)	128 (32.9%)		
	Postgraduate	781 (75.4%)	255 (24.6%)		
Mother's education	Secondary	65 (64.4%)	36 (35.6%)	12.648	0.002
	High school	276 (67.2%)	135 (32.8%)		
	Postgraduate	789 (75.1%)	262 (24.9%)		
						Paternal occupation	Employed	551 (77.5%)	160 (22.5%)	17.607	<0.001
Unemployed	579 (68.0%)	273 (32.0%)									
Maternal occupation	Employed	402 (75.4%)	131 (25.6%)	3.994	0.047
	Unemployed	728 (70.7%)	302 (29.3%)		
Smoking during pregnancy	No	1116 (72.8%)	418 (27.2%)	8.513	0.004
	Yes	14 (48.3%)	15 (51.7%)		
Neonatal disorders	No	1105 (72.5%)	419 (27.5%)	1.341	0.247
	Yes	25 (64.1%)	14 (35.9%)		
Birth weight	Low	116 (45.1%)	141 (54.9%)	113.321	<0.001
	Normal	984 (77.6%)	284 (22.4%)		
	High	30 (78.9%)	8 (21.1%)		
Season of birth	Spring	310 (72.8%)	116 (27.2%)	3.801	0.284
	Summer	293 (74.4%)	101 (25.6%)		
	Fall	264 (68.6%)	121 (31.4%)		
	Winter	263 (73.5%)	95 (26.5%)		

Predictors for ADHD

The significant variables in bivariate association (gender, school type, parental occupation and education, smoking during pregnancy, and low birth weight) were nominated to be entered into backward multiple binary logistic regression to investigate their contributions to ADHD. 

**Figure 1:**
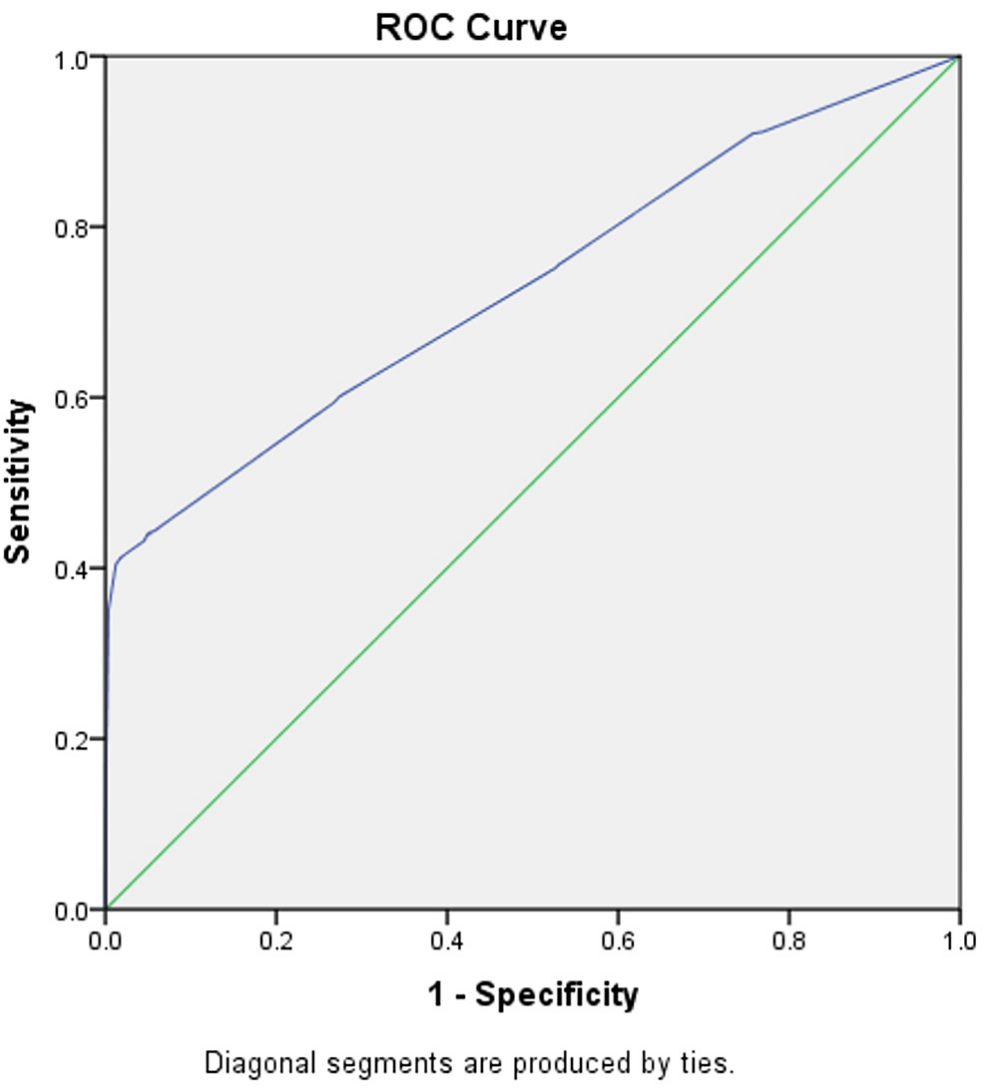
Receiver operating characteristic (ROC) curve

Four predictors out of eight were left over in the final model, with the amount of Nagelkerke R square 11.3% of ADHD variance explained by gender, school type, smoking during pregnancy, and birth weight. Moreover, the confusion matrix revealed that the overall model accuracy was 75.1%. The accuracy of the binary logistic regression model was also investigated using the receiver operating characteristic (ROC) curve (Figure 1). The findings showed that the model built using the entered variables successfully classified cases with an area under the curve (AUC) of 0.73 and a p-value of <0.001.

The data in Table 3 shows that the prevalence of ADHD was 1.5 times higher in public schools than in private ones, and that male children were 1.4 times more likely to have ADHD than female children. In addition, children of mothers who smoked cigarettes during pregnancy were also 2.4 times more likely to have ADHD than those of non-smoking mothers. Finally, children with a low birth weight were shown to be four times more likely to have ADHD than children with a normal birth weight. A child's age, parental occupation, and level of education did not correlate with ADHD symptoms in children.

**Table 3:** Logistic regression analysis of the significant risk factors B - unstandardized coefficient

Predictor	B	SE	Wald	p-value	Odds ratio	95% CI for odds ratio	
						Lower	Upper
School type (public vs. private)	0.380	0.122	9.740	0.002	1.463	1.152	1.858
Gender (male vs. female)	0.367	0.119	9.505	0.002	1.443	1.143	1.822
Smoking during pregnancy (yes vs. no)	0.879	0.395	4.955	0.026	2.410	1.111	5.227
Birth weight (low vs. normal)	1.384	0.145	91.423	0.000	3.990	3.005	5.298

Parent-teacher agreement levels toward Conners Rating Scale for ADHD diagnosis

This study allowed teachers and parents to evaluate the child's behavior to screen for ADHD. The intraclass correlation was used to measure the agreement between two raters on a scale variable level. Cohen's kappa was used for the agreement between two raters after classifying the children with or without ADHD based on a cut point of 15. The results showed that the intraclass correlation (ICC) with mixed-way effect and the absolute agreement was found to be 0.434, indicating that both raters (parents and teachers) had a low level of agreement. According to Cohen's kappa for dichotomous agreement, parents and teachers had a low agreement on classifying children as having positive or negative ADHD (kappa of 0.212).

## Discussion

ADHD is one of the most prevalent neurodevelopmental disorders among children, and it is frequently observed in school-aged students, which may provide an opportunity for early detection and management. 

Meta-analyses have found a wide range of conflicting estimates of the prevalence of ADHD [16]. According to Polanczyk et al.'s most recent meta-analysis study, which comprised 41 studies from 27 different countries, the worldwide prevalence of ADHD in children and adolescents is 3.4% [17]. According to a recent meta-analysis by Thomas et al. of 175 relevant papers from throughout the world, the worldwide prevalence of ADHD in children and adolescents is 7.2% [6,7]. 

According to our study, children had an ADHD prevalence of 27.7% according to parents and 22.5% according to teachers, which is within the range of prevalence of ADHD in 11 Arab countries [7,8]. 

In Jordan, only two studies with vastly different ADHD prevalence percentages have been published: one in the Al-Qasr area in the south of Jordan with a prevalence of 6.24%, studied on 4374 students according to a teacher's questionnaire and using the fourth edition of Diagnostic and Statistical Manual (DSM4) [18], and the other in Al-Mafraq in the north of Jordan with a prevalence of up to 40.62%, studied on 480 schoolchildren according to a teacher's perspective and using Attention Deficit Disorder Evaluation Scale (ADDES) [19]. 

A study conducted in Saudi Arabia revealed that the prevalence of ADHD ranged from 21.3% to 35.34%, according to the ADHD subtypes [7]. Another study in Egypt reported that the prevalence was 20.9% [20]. However, it is important to note that there is no agreement on the prevalence rates and that studies conducted in different parts of the world find substantial differences in these percentages. This vast variation could be explained by the method of assessment and informants, the type of sample collected, sample size, age group, and social and cultural factors [8,21]. 

The present study documented that being male was significantly correlated with ADHD, which is similar to international literature [22], although the gender gap has been decreasing, which could be explained by the early detection of ADHD symptoms in girls [10]. 

Similar to Langley et al.'s study [23], our study found that smoking during pregnancy and low birth weight were linked to ADHD. Regarding the parents' education, we found a significant correlation with ADHD in bivariate correlation, which is consistent with a study conducted in Norway that revealed that children whose parents did not complete high school had a roughly fourfold increased probability of having severe symptoms [24] and another study conducted in China that reported an increased risk of ADHD [25]. In contrast, Al Azzam et al.'s study in Jordan and Al Ghannami et al.'s study in Oman found no link between parental education and ADHD [19,26]. In agreement with a study done in Denmark, we found an association between unemployed parents and ADHD [27]. 

In contrast to our findings regarding neonatal disorders, a study conducted in China revealed that during their growing stage, infants with jaundice were at high risk for having ADHD, as determined by a physician [28]. Season of birth was not considered in our study as a risk factor for ADHD; this is inconsistent with a longitudinal study done by Zhang et al., which showed that babies born in the spring season were more likely to have ADHD [29]. 

According to our data, we found low agreement between parent and teacher scales since it's a subjective measurement, which is consistent with a study done in the United Arab Emirates [15]. 

This study is the first in Jordan to use the 10-item Conners rating scale, which is a brief and valid instrument for assessing and screening for ADHD [30], and to use both the parent and teacher forms of the Conners rating scale, whereas two previous studies in Jordan used only the teacher form. 

Limitations

This research was only carried out in Amman; therefore, the extent to which its findings can be generalized is restricted. Another limitation is that there was no clinical interview to verify the ADHD diagnosis. 

## Conclusions

ADHD is a highly prevalent problem among primary school children in Jordan. The prevalence was found to be higher than the international values. To date, this is the first study in Jordan to focus on crucial risk factors such as smoking during pregnancy, birth weight, and parental occupation, as well as to involve both parents and teachers as informants to assess the prevalence of ADHD. 

These findings underline the importance of increasing ADHD assessment and monitoring among schoolchildren from various socioeconomic backgrounds as well as dealing with modifiable risk factors. They also emphasize the significance of obtaining correct diagnoses and providing culturally appropriate care. 
